# E-cigarette addiction and harm perception: Does initiation flavor choice matter?

**DOI:** 10.1186/s12889-022-14166-w

**Published:** 2022-09-20

**Authors:** Man Hung, Andrew Spencer, Eric S. Hon, Frank W. Licari, Val Joseph Cheever, Ryan Moffat, Clarissa Goh, Ben Raymond, Martin S. Lipsky

**Affiliations:** 1 Roseman University of Health Sciences College of Dental Medicine, 10894 S. River Front Parkway, South Jordan, Utah 84905 USA; 2 University of Utah School of Medicine, Salt Lake City, UT USA; 3 Emory University, Atlanta, GA USA; 4 University of Chicago Department of Economics, Chicago, IL USA; 5 Riverton High School, Riverton, UT USA; 6 Portland State University Institute on Aging, Portland, OR USA

**Keywords:** PATH, Electronic cigarette, Addiction, Harm perception, Vaping, Smoking, Flavors, Word count: 3,045

## Abstract

**Introduction:**

The 21^st^ century was marked by a dramatic increase in adolescent e-cigarette use in the United States (US). The popularity of non-traditional flavor types, including fruit and pastry, is thought to contribute toward growing product use nationally, leading to a variety of federal and state regulations limiting the use of non-traditional flavors in the US. The relationship between flavor type and increased adolescent use suggests a possible link between flavor use and addiction and harm perception. This study assessed if the flavor type used when initiating e-cigarette use predicted addiction and harm perceptions.

**Methods:**

The study utilized data from the multi-wave youth Population Assessment of Tobacco Health Study. It explored the impact initiating e-cigarette use with traditional versus non-traditional flavor types among cigarette users on the outcome variables: e-cigarette addiction and harm perception. Both e-cigarette addiction and harm perception were measured using self-report, Likert scale questionnaires. Descriptive statistics characterized the study variables and linear regression analyses performed to test whether flavor initiation type is associated with addiction and harm perception.

**Results:**

The study sample consisted of 1,043 youth (weighted *N* = 1,873,617) aged 12 to 17 years who reported at least one instance of e-cigarette use. After adjusting for age, age of onset, sex, race and annual household income there was no statistically significant difference in addiction levels between those initiating with traditional versus non-traditional flavors (*p* = 0.294). Similarly, traditional versus non-traditional flavor initiation did not show a statistically significant difference in adolescent e-cigarette harm perceptions (*p* = 0.601).

**Conclusions:**

Traditionally flavored e-cigarette initiation produces similar risk for addiction and harm perceptions as non-traditionally flavored initiation. These findings suggest that banning non-traditional flavors alone may be ineffective in curbing e-cigarette addiction and harm perception. Additional research is needed to better understand which e-cigarette product characteristics and behaviors may be associated with greater addiction and reduced harm perceptions.

## Background

With the decline in use of cigarettes and other combustible tobacco products in the United States (US) [1], many experts hoped that tobacco products would fade from popular use by the next generation of adolescents. Unfortunately, electronic cigarettes, one of the many forms of electronic nicotine delivery systems (ENDS) have introduced millions of current youth to a new tobacco product often at exceedingly young ages. E-cigarettes come in a wide variety of flavors and product designs, which enhances their appeal, particularly amongst adolescents [2–5].

While the harmful effects of cigarettes and other combustible products are robust and well-documented [6], e-cigarettes have not been on the market long enough for comprehensive research to assess their potential negative long-term health effects. E-cigarette manufacturers consistently claim that their products are a healthier alternative to combustible tobacco products [7], but researchers and medical professionals challenge this assertion, citing a lack of research evaluating safety and therefore encouraging adolescents to avoid e-cigarette use [8]. Despite this, e-cigarettes continue to grow in popularity among adolescents. In the United States in 2021, approximately 2.06 million youth reported e-cigarette use, including 11.3% of high school students and 2.8% of middle school students. Daily use was reported in 27.6% of current high school users and 8.3% of current middle school users [9, 10].

One aspect of e-cigarette product appeal to adolescents may be the wide range of flavor types. First generation e-cigarettes typically mirrored the traditional cigarette experience with a less sophisticated design and a smaller variety of flavors such as tobacco, menthol, and mint that simulated the popular cigarette flavors on the market [11]. Over time, e-cigarette companies began to manufacture new flavors, such as fruit, pastry, clove, alcoholic drink, and soda, which quickly garnered popularity among younger, non-cigarette smokers [11]. A recent review of adolescent e-cigarette initiation patterns estimated that 81% of young e-cigarette users started with a non-traditional flavor [12]. Corroborating this, a study of California high school students found that among students currently using e-cigarettes, over 90% used non-traditionally flavored products [13, 14].

Several other studies examining e-cigarettes also identified non-traditional flavors as a reason for continued adolescent e-cigarette use [3, 15, 16]. A 2014 survey found that 81% of adolescent users cited flavor availability as their primary reason for using e-cigarettes [17, 18]. To address the link between novel flavors and e-cigarette use in adolescents, the US government banned the sale of cartridge-based e-cigarettes containing flavors other than menthol and tobacco in early 2020 [19]. Although the Food and Drug Administration enforced this policy on non-traditional flavors, the ban only applied to pre-filled, cartridge-based products and allowed the sale of other product types, such as re-fillable cartridges, to continue with non-traditional flavor types [20, 21]. While non-traditional flavor may remain available on a limited basis, evidence suggests that many adolescents responded to the flavor ban by simply switching to an available flavor. Research on adolescent e-cigarette use patterns before and after the national ban found that menthol flavor sales increased from 10.7% to 61.8%.

A key chemical component of e-cigarettes is nicotine, a substance known to cause addiction [22]. Addiction is characterized by a perceived loss of control in product use [23] and is typically measured along several dimensions, including craving, affiliative attachment, loss of control, and affective enhancement [24, 25]. Higher levels of e-cigarette addiction are linked with continued use [26]. Most past research on adolescent e-cigarette addiction compares e-cigarette versus cigarette addiction susceptibility but does not examine whether e-cigarette flavorings and other characteristics foster an increased susceptibility to addiction [13, 14, 17, 18, 27]. This study sought to assess the potential role of e-cigarette flavor initiation type on addiction.

In addition, youth harm perception of e-cigarettes merits further research [28, 29]. An estimated one-third of US adolescents perceive e-cigarettes as less harmful than cigarettes. Among current e-cigarette users, this increases to three-fourth of users [28] and e-cigarette harm perception predicts subsequent use in following years [30]. Research implicates flavor as playing a role in an adolescent’s perception of e-cigarette harm with adolescents perceiving fruit flavored e-cigarette products as less harmful than menthol and tobacco flavors [31]. Thus, a second study aim was to examine the relationship between e-cigarette flavor initiation type and harm perception in adolescents. We hypothesize that adolescent non-traditional e-cigarette flavor initiation will be associated with an increased likelihood of future addiction and reduced perceptions of product harm.

## Methods

### Data Source

This study used data from the Population Assessment of Tobacco and Health (PATH) Study [32]. The PATH Study is collaboratively sponsored by the National Institute of Drug Abuse, National Institute of Health, Center for Tobacco Products, and Food and Drug Administration. It consists of longitudinal interview and self-reported survey questions using audio computer-assisted self-interviews administered in English or Spanish to parents, adults, and youth pertaining to tobacco use, behavior, attitudes, beliefs, and health outcomes. It collected data bi-annually in five waves (1,2,3,4 and 4.5) from 2011 to 2019, using weighting procedures to adjust for oversampling and nonresponse which were then further adjusted based on US Census Bureau data to develop a nationally representative study group. About 46,000 people aged 12 years and older, including tobacco users and non-users, were included in the first wave of the PATH Study and followed over time. This study utilized longitudinal data from waves 2, 3, 4, and 4.5 of the PATH Study databases among cigarette users. The wording of wave 1’s questions were less specific and differed from subsequent waves and wave 1’s data was thus excluded from this study. More details regarding PATH can be found at https://www.drugabuse.gov/research/nida-research-programs-activities/population-assessment-tobacco-health-path-study.

### Measures

#### Demographics

The sociodemographic variables included participant age, gender, race, ethnicity, grade level, age of cigarette smoking initiation, and household income from wave 4.5. Wave 4.5 was selected because it contained the most current data for PATH youth participants.

#### Outcome Measures

The research team reviewed the PATH database and selected six questions related to e-cigarette addiction and three questions related to e-cigarette harm perceptions as outcome measures. Outcome scores were solely derived from the most PATH Wave 4.5. E-cigarette initiation flavor was derived from Wave 2,3,4, or 4.5, depending on when the respondent first reported e-cigarette usage. Only participants who remained in the study from their first reported use of e-cigarettes to the most recent wave were included in the analysis.

##### E-Cigarette Addiction

Measures of e-cigarette addiction came from wave 4.5 in which participants reported their level of agreement on six variables (i.e., items): (1) I find myself reaching for electronic nicotine products without thinking about it, (2) Frequently crave electronic nicotine products, (3) My electronic nicotine product use is out of control, (4) Using electronic nicotine products helps me feel better if I've been feeling down, (5) Using electronic nicotine products helps me think better, and (6) I would feel alone without my electronic nicotine products. The response options for all six items used a 5-point Likert scale which ranged from 1 (not at all true) to 5 (extremely true).

##### E-Cigarette Harm Perception

Measures of harm perception came from wave 4.5 data in which participants responded to the following three items: (1) Harmfulness of electronic nicotine products to health (Response options: 1=Not at all, 2=Slightly, 3=Somewhat, 4=Very, 5-Extremely), (2) Thoughts on how much people harm themselves when they use e-cigarettes or other electronic nicotine products (Response options: 1=No harm, 2=Little harm, 3=Some harm, 4=A lot of harm), and (3) Harmfulness of using e-cigarettes or other electronic nicotine products compared to smoking cigarettes (Response options: 1=Less harmful, 2=About the same, 3=More harmful).

#### Predictor

The predictor was e-cigarette flavor type initiation. Measures about the e-cigarette flavor type used at initiation came from waves 2, 3, 4 and 4.5 of the PATH Study depending on when participants reported previous use of e-cigarettes. Only participants reporting previous ENDS use answered questions about the flavor type initiation. The study examined two general types (traditional and non-traditional) of e-cigarette flavor initiation. Traditional types included standard tobacco, menthol, or mint flavors. Non-traditional types included fruit, clove/spice, alcoholic drink, non-alcoholic drink, and candy/dessert/other sweets. The study excluded respondents that selected more than one initiation flavor type.

#### Covariates

Sociodemographic factors such as age, sex, race and annual household income, and the age at which they started smoking cigarettes regularly can impact e-cigarette addiction and harm perception [28, 33, 34]. Therefore, this study adjusted for the effects of these covariates in statistical analyses. The survey asked participants to quantify an estimate for the total number of instances they had used an e-cigarette and similarly estimate the age at which they initiated e-cigarette use. The statistical analysis controlled for these estimates.

### Statistical Approach

Data analyses were conducted using SPSS for Windows version 28. Descriptive statistics characterized the study sample. Frequency distributions of e-cigarette flavor initiation type of both unweighted and weighted frequencies and proportions were computed and reported. The weighted values were derived from the all-wave youth cohort file and represent national population estimates while unweighted numbers represent sample estimates. Even though the sample is large, it may not accurately represent the entire US without adjusting the sample to represent the population.

Exploratory factor analysis was performed on the six addiction and three harm perception items to assess the factor structure of the items using principal axis factoring and varimax rotation with Kaiser normalization. Investigating the factor structure of items determines whether items associate with each other to form a latent construct (e.g., factor). If the six addiction items have similar patterns of item responses, they will measure the underlying latent construct of addiction and can be used to generate a composite score (e.g., factor score) for analyzing addiction. This facilitates interpretation, since the outcome measures of addiction as a whole is of greater interest than the outcome of each individual addiction item [35].

Factor loading evaluates factor structure and determines how strongly items fit or associate with each other to form one underlying construct. It weighs the correlation of an item with the construct. Factor loading values range from -1 to 1, with values larger than |0.4| regarded as being relevant and having adequate fit for a construct [36]. The Kaiser-Meyer-Olkin Measure of Sampling Adequacy tests for the sampling adequacy of the selected items and the complete dataset. Using this method, a value of >0.6 indicates that factor analysis could be applied to the dataset and a Barlett’s test of sphericity with *p<0.05* shows that the selected items were correlated. More detailed descriptions of factor analysis can be found elsewhere [37].

The reliabilities of the addiction factor and harm perception factor were then examined using Cronbach’s alpha with a satisfactory Cronbach’s alpha value set > 0.6 [38]. Cronbach alpha values range from 0 to 1 with larger values representing greater reliability [39]. After each factor demonstrated adequate factor loadings for its items and adequate reliability, composite (factor) scores for both the addiction and harm perception outcomes were created using a linear scale metric. Higher factor scores signified that they had higher levels of addiction and perceived the products as more harmful. Factor scores are essentially a standardized, weighted average of the items’ scores, with the items’ weights coming from the factor loadings. Since most items have unequal correlations with an underlying construct, average item scores should not be used to represent a construct. Using factor scores more appropriately reflects the strength of association with different items.

Linear regression analyses of the composite scores (e.g., factor scores) for addiction and harm perception were used to examine the two research questions: (1) Does e-cigarette flavor initiation type predict e-cigarette addiction, with and without adjustment for a person’s age, age when they first started smoking cigarettes regularly, sex, race and annual household income? (2) Does e-cigarette flavor initiation type predict e-cigarette harm perception, with and without adjustment for a person’s age, age when they first started smoking cigarettes regularly, sex, race and annual household income? The standardized regression coefficient with an associated 95% confidence interval and R [2] were calculated. A two-tailed *p*-value < 0.05 was considered statistically significant.

## Results

The study sample consisted of 1,043 adolescent participants (weighted N = 1,873,617) aged 12 to 17 years old from PATH Wave 4.5. Among the group, 52.6% were male, 77.9% were White, 24.6% were Hispanic, and 21.1% were between 12 and 14 years old. Table 1 summarizes the demographic characteristics of the study group. Among the sample group, 5.6% were under 12 years old when they first started smoking e-cigarettes, 39.6% had an annual household income of more than $100,000, and nearly 80% had a parent/spouse/guardian with some college education or above. About 16% of the sample, representing more than 300,000 US adolescents, initiated e-cigarette smoking with a traditional flavor (e.g., tobacco flavor or the menthol/mint flavor). In contrast, 84 % of the sample representing 1,573,345 US adolescent-initiated e-cigarette usage with a non-traditional flavor such as clove spice, fruit, chocolate, non-alcoholic drink, dessert or other flavor. Table 2 displays flavor choices by sample size and percentage, with the weighted sample size representing the US adolescent population.

**Table 1:** Descriptive statistics of demographics and outcome variables

Variable	Mean (SD)	Min/Max	unweighted n (%)	weighted n (%)
Age				
12 to 14 years old			220 (21.1)	340825 (18.2)
15 to 17 years old			823 (78.9)	1532792 (81.8)
Sex				
Male			546 (52.6)	965544 (51.5)
Female			493 (47.4)	903587 (48.2)
Race				
White			776 (77.9)	1456827 (80.6)
Black			78 (7.8)	122904 (6.8)
Other			142 (14.3)	227342 (12.6)
Ethnicity				
Hispanic			249 (24.6)	327145 (18.1)
Non-Hispanic			764 (75.4)	1483123 (81.9)
Grade level				
<=7th grade			31 (3.0)	42924 (2.3)
8th grade			74 (7.1)	137834 (7.4)
9th grade			158 (15.2)	252747 (13.5)
10th grade			248 (23.8)	448227 (24.0)
11th grade			259 (24.9)	471746 (25.2)
Other			271 (25.0)	515356 (27.6)
Age when first started smoking cigarettes regularly				
<12 years old			3 (5.7)	4335 (5.6)
12 to 14 years old			19 (35.8)	24799 (32.1)
15 to 17 years old			31 (58.5)	48158 (62.3)
Number of times used ENDS in life				
1			336 (34.1)	584939 (33.2)
2-10			290 (29.4)	551272 (31.3)
11-20			109 (11.1)	184286 (10.4)
21-50			94 (9.5)	174160 (9.9)
51-99			42 (4.3)	80164 (4.5)
100			114 (11.6)	189234 (10.7)
Anyone lives with you now uses tobacco				
Cigarettes, cigars, cigarillos or filtered cigars			311 (30.0)	509271 (27.3)
E-products exclusively			73 (7.1)	127175 (6.8)
Other tobacco products			60 (5.8)	96586 (5.2)
No one living in the home uses tobacco			591 (57.1)	1129037 (60.6)
Parent/guardian marital status				
Married			642 (62.6)	1207778 (65.7)
Widowed, divorced or separated			272 (26.5)	466650 (25.4)
Never married			112 (10.9)	163995 (8.9)
Annual household income				
<$10,000			40 (4.0)	48798 (2.7)
$10,000 to $24,999			130 (13.0)	199276 (11.1)
$25,000 to $49,999			204 (20.4)	337605 (18.8)
$50,000 to $99,999			271 (27.1)	499916 (27.8)
>=$100,000			355 (35.5)	712960 (39.6)
Parent/spouse/guardian educational level				
Less than high school			71 (6.9)	105673 (5.7)
GED			32 (3.1)	46559 (2.5)
High school graduate			156 (15.2)	235975 (12.8)
Some college or associate degree			307 (29.9)	557953 (30.3)
Bachelor’s degree			247 (24.1)	468069 (25.5)
Advanced degree			213 (20.8)	424193 (23.1)
Addiction score	0.438 (0.933)	0.000/6.466		
Harm perception score	1.934 (0.905)	0.000/3.602

**Table 2:** Distribution of various e-cigarette flavor initiation type (Total unweighted *N* = 1,043; Total weighted *N* = 1,873,617).

Variable	Unweighted N (%)	Weighted N (%)
**Traditional flavor initiation type**	**170 (16.30)**	**300272 (16.03)**
Tobacco	37 (3.55)	59891 (3.20)
Menthol/Mint	133 (12.75)	240381 (12.83)
**Non-Traditional flavor initiation type**	**873 (83.70)**	**1573345 (83.97)**
Clove Spice	5 (0.48)	5417 (0.29)
Fruit	571 (54.75)	993689 (53.04)
Chocolate	9 (0.86)	16311 (0.87)
Alcohol	6 (0.58)	8195 (0.44)
Non-alcoholic drink	34 (3.26)	57488 (3.07)
Dessert	223 (21.38)	437271 (23.34)
Other flavor	25 (2.40)	54974 (2.93)

Table 3 shows the item response distribution of addiction and harm perception items. Analyzing the six addiction and three harm perception items revealed sampling adequacy and reliable estimates for both the addiction factor (Kaiser-Meyer-Olkin Measure of Sampling Adequacy = 0.854, Bartlett’s test of sphericity value *p* < 0.05) and for the harm perception factor (Kaiser-Meyer-Olkin Measure of Sampling Adequacy = 0.649, Bartlett’s test of sphericity value *p* < 0.05). This indicates that the sample is adequate for conducting exploratory factor analysis. The factor loadings for the addiction items ranged from 0.692 to 0.794, with a Cronbach’s alpha of 0.852. For the three harm perception items, the factor loadings ranged from 0.540 to 0.836 with a Cronbach alpha of 0.743. These results provided empirical support for calculating a composite factor score for both addiction and harm perception. Table 4 displays the factor loading values of the addiction and harm perception items.

**Table 3:** Distribution of e-cigarette addiction and harm perception item responses.

	Mean	Median	Std Dev	Min	Max
E-cigarette addiction					
(1) Reach for product	1.40	1.00	0.870	1	5
(2) Frequent crave	1.27	1.00	0.730	1	5
(3) Out of control	1.13	1.00	0.550	1	5
(4) Help feel better	1.51	1.00	1.015	1	5
(5) Help think better	1.29	1.00	0.748	1	5
(6) Alone without product	1.13	1.00	0.545	1	5
E-cigarette harm perception					
(1) Harmfulness Nicotine	3.23	3.00	1.119	1	5
(2) Overall harmfulness	2.82	3.00	0.833	1	4
(3) Harm ENDS vs. CIGS	1.52	1.00	0.611	1	3

**Table 4:** Factor structures of e-cigarette addiction and harm perception.

	Factor 1 loading	Factor 2 loading
E-cigarette addiction		
(1) Reach for product	0.692	-0.074
(2) Frequent crave	0.794	-0.053
(3) Out of control	0.726	0.036
(4) Help feel better	0.692	-0.141
(5) Help think better	0.718	-0.118
(6) Alone without product	0.726	-0.009
E-cigarette harm perception		
(1) Harmfulness Nicotine	-0.129	0.836
(2) Overall harmfulness	-0.073	0.773
(3) Harm ENDS vs. CIGS	0.002	0.540

After adjusting for covariates in the multivariate linear regression model, e-cigarette addiction levels when an adolescent-initiated e-cigarette smoking with traditional flavors as opposed to non-traditional flavors were not statistically significant (*B* = -0.163; 95% CI = -1.285 to 0.398; R [2] = 0.444; *p* = 0.294) (Table 5). Additionally, e-cigarette initiation with traditional flavors contributed similarly as the perception of harm than non-traditional flavors (*B* = 0.082; 95% CI = -0.685 to 1.169; R [2] = 0.423; *p* = 0.601) (Table 5).

**Table 5:** Linear regression analyses predicting e-cigarette addiction and harm perception from e-cigarette flavor initiation type (with and without adjustment for age, age when first started smoking cigarettes regularly, sex, race and annual household income).

Variable	*B* [95% CI]^b^	*p*-value^b^	*B* [95% CI]^c^	*p*-value^c^
E-cigarette addiction				
Traditional flavor type initiation^a^	-0.110 [-0.182, 0.125]	0.718	-0.163 [-1.286, 0.398]	0.294
Age^a^			0.281 [-0.371, 1.766]	0.060
Age when first started smoking cigarettes regularly			-0.388 [-1.123, -0.153]	0.011
Sex^a^			-0.163 [-0.949, 0.261]	0.258
Race^a^			-0.085 [-0.908, 0.498]	0.559
Annual household income			0.079 [-0.215, 0.369]	0.599
E-cigarette harm perception				
Traditional flavor type initiation^a^	0.077 [0.040, 0.338]	0.013	0.082 [-0.685, 1.169]	0.601
Age^a^			0.251 [-0.153, 1.832]	0.095
Age when first started smoking cigarettes regularly			-0.022 [-0.572, 0.495]	0.884
Sex^a^			0.203 [-0.200, 1.132]	0.166
Race^a^			0.100 [-0.511, 1.037]	0.496
Annual household income			-0.207 [-0.540, 0.104]	0.179

^a^Reference is non-traditional flavor type initiation, age 12 to 14 years old, male and Non-White.

^b^Without adjustment.

^c^With adjustment.

## Discussion

Initially touted by proponents as a safer alternative to conventional combustible tobacco products, critics point out that e-cigarettes pose their own unique harms [40]. While e-cigarettes may be somewhat helpful as a smoking cessation aid [41, 42], they also promote dual use with combustible products and entice youth into using tobacco products. Adolescents report flavor as a common incentive for trying and continuing to use e-cigarettes. To our knowledge, this study is the first to develop composite scores from national survey items in order to examine whether e-cigarette flavor initiation type is associated with e-cigarette addiction and harm perception among US adolescents.

After adjusting for covariates, this study found no statistically significant difference in addiction outcomes between youth initiating e-cigarette use with traditional instead of non-traditional flavors. Similarly, after adjusting for covariates, no difference between the two groups emerged in harm perception. These findings suggest flavor initiation has no association with either addiction or harm perceptions. Unexpectedly, these results contradict an earlier study by Landry et al. which reported significantly higher rates of perceived addiction among flavored e-cigarette users over non-flavored e-cigarettes users [2]. However, this earlier study used a sample that could not be generalized to the larger US population and focused on adult users rather than adolescents.

One explanation for the lack of difference may be the inclusion of menthol/mint flavor to traditional flavors. Menthol/mint may impact the findings since it is associated with adolescent smoking behaviors and augments nicotine addiction [43, 44]. Furthermore, the study looked only at those initiating use and not continued users. Nonetheless, the finding that perceived addiction among youth did not differ between those initiating use with traditional versus non-traditional flavored products is valuable for policy makers to consider. From 2011 to 2018, adolescent use of e-cigarettes in the US increased by 1800% [45] and about 1 in 4 high school students reported e-cigarette use [46]. E-cigarettes serve as a gateway to combustible smoking. Compared to those who have never tried an e-cigarette, young people in the US who have tried e-cigarettes have far greater odds of trying cigarettes and an eight times greater risk of using cigarettes one year later. Seeking to curb this dramatic growth, US regulators banned non-traditional flavors. Yet the finding that perceived addiction was not greater among those initiating non-traditional flavored e-cigarettes suggests that this ban may be ineffective in curtailing e-cigarette use.

Perception of harm is also a predictor of future and continued e-cigarette use [28, 47]. In accordance, it is paramount that policy makers and the medical community understand differences in harm perceptions. Although considered safer than combustible products [48], toxicology studies demonstrate the potential adverse impact of e-cigarettes on the respiratory, cardiovascular, and immune systems and the long-term effects remain unknown [49, 50]. Given the similar levels of perceived addiction and harm, failure to address use of traditional flavors alongside use of non-traditional flavors may not lower the prevalence of e-cigarette use. To effectively curb e-cigarette use and reduce their health impact, future legislation will need to address traditional flavor types like menthol and tobacco. Further research is needed to examine the benefit of banning non-traditional flavors.

In addition to harm perception and addiction, two noteworthy sample characteristics emerged. Among the adolescents who used e-cigarettes, nearly 80% of their parents/guardians had some college education or above. This suggests that alongside increased regulation, improving the health literacy of parents about the harms and allure of e-cigarettes may be effective in reducing use. Furthermore, over 5% of e-cigarette users were less than 12 years old when they first started vaping, highlighting the need for initiating prevention strategies at an early age.

### Limitations

The current study has several limitations. First, the PATH Study data were self-reported and potentially subject to bias. Respondents might answer with what they believe to be the most acceptable answer rather than the truth. This study assessed perceived addiction, a subjective measurement, but variables (e.g., social and peer pressure, advertising by manufacturers, modeling by famous people, geographic and financial accessibility) can modify perception so that it may not accurately reflect addiction. Furthermore, menthol and mint flavors were included within the same variable, despite popular manufacturers such as Juul producing separate menthol and mint flavor categories [20, 21]. This precluded separately assessing menthol flavor users from mint flavor users. Since evidence connects menthol with vaping satisfaction and perceived addiction [2], research examining its use independently is valuable to see if perceptions of addiction and harm differ for this flavor [20, 21].

### Implications

The initiation of e-cigarette product use among adolescents with traditional flavors poses similar perceptions for addiction and harm as non-traditional flavors. These findings can guide policy makers and suggest that banning flavored products alone may fail to significantly reduce e-cigarette use. Additional research is needed to better understand which e-cigarette product characteristics and behaviors lead to increased risk for product dependence and successful conveyance of the harms of e-cigarettes.

## Data Availability

All of the data used in this study were fully available at https://www.icpsr.umich.edu/web/NAHDAP/studies/36498/datadocumentation.
